# An Insight in Developing Carrier-Free Immobilized Enzymes

**DOI:** 10.3389/fbioe.2022.794411

**Published:** 2022-03-02

**Authors:** Vivek Chauhan, Diksha Kaushal, Vivek Kumar Dhiman, Shamsher Singh Kanwar, Devendra Singh, Vinay Kumar Dhiman, Himanshu Pandey

**Affiliations:** ^1^ Department of Biotechnology, Himachal Pradesh University, Shimla, India; ^2^ B.N. College of Engineering and Technology, Lucknow, India; ^3^ Dr. Y. S. Parmar University of Horticulture and Forestry Nauni, Solan, India

**Keywords:** enzymes, immobilized enzymes, biocatalytic process, carrier-free immobilized enzymes, immobilization

## Abstract

Enzymes play vital roles in all organisms. The enzymatic process is progressively at its peak, mainly for producing biochemical products with a higher value. The immobilization of enzymes can sometimes tremendously improve the outcome of biocatalytic processes, making the product(s) relatively pure and economical. Carrier-free immobilized enzymes can increase the yield of the product and the stability of the enzyme in biocatalysis. Immobilized enzymes are easier to purify. Due to these varied advantages, researchers are tempted to explore carrier-free methods used for the immobilization of enzymes. In this review article, we have discussed various aspects of enzyme immobilization, approaches followed to design a process used for immobilization of an enzyme and the advantages and disadvantages of various common processes used for enzyme immobilization.

## Introduction

Enzymes have been the most important part of our day-to-day life. Enzymes can regulate the biochemical and chemical reactions in the organisms as well as *in situ* biotransformations without being altered in the process (Palmer and Bonner, 2007). These biocatalysts are mostly used in the industries such as pharmaceutical and dairy industries for making food and dairy products, pharmaceutical industries for making medicines, textile industry for texture improvement, and paper and pulp industry (Watanabe et al., 1988; Sheldon and Woodley, 2018). To increase the use of enzymes on an industrial scale as biocatalysts (Zhang et al., 2012; Chapman et al., 2018), it is mandated that the enzyme system must be stable in a reaction system, the enzyme must possess improved operational stability in an aqueous or organic or biphasic system, stable biocatalytic potential, optimal requirement of raw materials, and more, so the enzyme selectivity and specificity should be high (Kricka and Thorpe, 1986). On top of a biocatalytic system, preferably, the enzyme system should be driven into a hygienic and cleaner industrial process.

The goal of enzyme immobilization is to create a strong biocatalyst that can operate under non-native and severe conditions for a longer period. Therefore, instead of using soluble enzyme counterparts, countless efforts have been committed for the improvement of enzyme immobilization techniques, optimizing their catalytic efficiency for a greater yield, stability, and reusability (Cao, 2005; Cao et al., 2021). For instance, it is recommendable to identify a suitable reusable matrix with a better selective absorbent, create a green recyclable process in order to improve the control of the catalysis process, and reduce the manufacturing cost of the desired product. Immobilized enzymes comprise two essential functional units irrespective of their methods of preparation or nature of the enzyme preparation: First, they possess a non-catalytic unit which is essential for their separation with the host environment, recycle process, and overall process management; Second, the functional catalytic unit which converts the substrate into a product (Cao, 2011; Dwevedi, 2016). Non-catalytic units comprise chemical and physical characteristics of the immobilized enzyme system, such as size, shape, and length of the chosen carrier, whereas the catalytic units are more similar to the chemical properties such as selectivity, pH, and activity (Cao et al., 2003). These are the criteria of choice when planning for the immobilization process of an enzyme. Carrier-free enzymes provide a cost-effective, simple, and straightforward method of reusing enzymes while also maintaining their catalytic efficiency and thermostability. This immobilization technology has been thoroughly evaluated upon numerous enzymes, and it has been successfully used in industrial processes (Velasco-Lozano et al., 2016). Thus, this review shows different aspects of enzyme immobilization and various approaches, along with the disadvantages and advantages of common processes used for enzyme immobilization.

## Approach Toward Immobilization of Enzymes

Currently, the use of enzymes as a robust immobilized biocatalyst system(s) is experiencing a passage through crucial transition(s). This is supported by the fact that the strategies used for the layout of immobilized enzymes have come to be more and more rational; often, more complex and advanced immobilization strategies are utilized to overcome difficulties of older immobilization strategies involving only a particular immobilization methodology (Cao, 2011). In this context, this article tries not only to summarize the plenty of the artwork in enzyme immobilization techniques but also examines the fashion of improvement of biocatalyst efficacy, the aggregate of numerous immobilization methods, strategies, or disciplines, which have been previously successfully employed to attain the favored.

## Immobilization of Enzymes and Old vs. New Strategies

During earlier times, the enzyme immobilization or in-solubilization process was synonymously used (Patel et al., 1969). The term “enzyme immobilization” refers to the physical confinement of the soluble proteinaceous enzyme molecules via different interactions to the carrier’s matrix in a region of space such as cross-linking/embedding, generally an insoluble material that can be easily removed from the medium, using simple basic procedures such as filtration, centrifugation, self-aggregation, or sieving (Mosbach, 1976). The characteristics of immobilized enzymes are largely governed by four important factors in an enzyme immobilization process, which are the nature and type of enzyme employed, the nature of the carrier, and the immobilization conditions (Datta et al., 2013; Liu et al., 2020).

To date, enzyme immobilization techniques have been extensively researched, with more than 6,000 publications and patents over it. Enzymes including amino acylase, PGA, invertase, many lipases, proteases, amylase, and nitrilase have been immobilized and are employed for diverse commercial processes (Heinen et al., 2017; Almeida et al., 2018; Facchini et al., 2018; Monteiro et al., 2019). Although the primary techniques of enzyme immobilization may be classified into some special techniques only, yet covalent bonding, adsorption, entrapment, encapsulation, and cross-linking are all examples of modifications that have been produced in the past, largely based on combinations of authentic techniques (Ahmad and Sardar, 2015). Similarly, numerous carriers of varied physical and chemical natures or occurrences have been developed for a wide range of bio-immobilization and bio-separation media. It is critical to remember that none of the existing immobilization methods can tackle the challenges that will be encountered in a certain process when building the best-suited immobilized enzyme for that process (Cao, 2011). Optimization and stabilization can be additionally carried out with the aid of a chemical change. One of the often used strategies to enhance the enzyme balance is hydrophilization of enzyme molecules through chemical change with hydrophilic practical polymers. The stabilization impact due to hydrophilization of a selected enzyme is manifested because of the creation of a positive hydrophilic microenvironment. The entrapment of the stabilized enzyme frequently results in the formation of an extra strong immobilized biocatalyst in comparison with an entrapped biocatalyst (Cao, 2005).

In the older case, the entrapped enzymes might be in addition subjected to chemical cross-linking to improve the balance or avoidance of enzyme leakage. Remarkably, a *β*-amylase obtained from *Bacillus megaterium*, immobilized in a bovine serum albumin gel matrix and covalently cross-connected depicted a 14-fold better thermostability than that of a native enzyme (Ray et al., 1994). Generally, the combination of techniques, including the pre-immobilization strategies, together with imprinting, chemical modification, cross-linking, etc., with the right immobilization method is decided as an essential immobilization technique (Cao, 2011).

**FIGURE 1:**
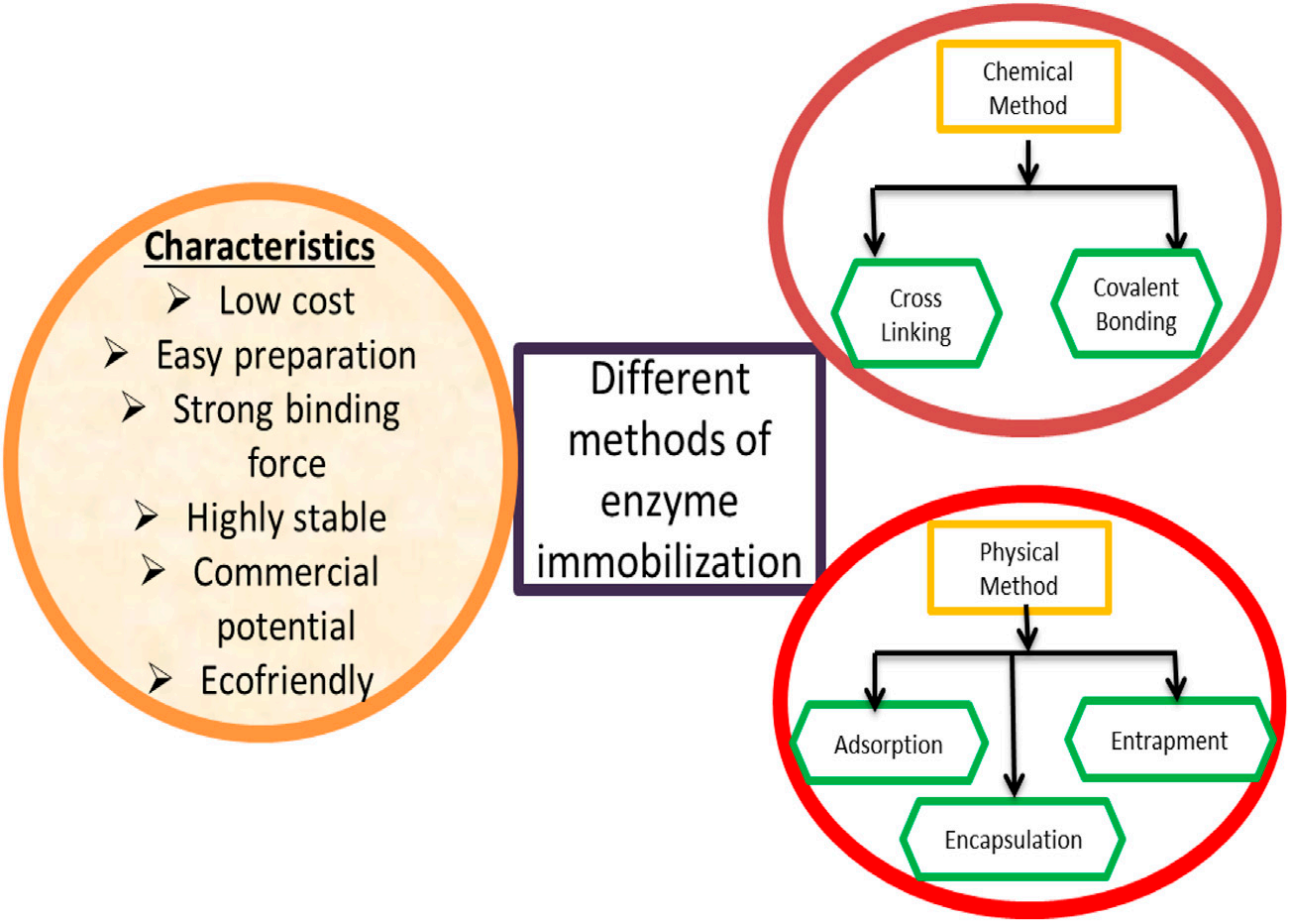
Chemical and physical method(s) of immobilization and their characteristics.

When designing an immobilized enzyme for any biological process, it is far critical to take note of the truth that the selection of the immobilization techniques is very important to get the desired results (Mohamad et al., 2015). The practical method would possibly be the usage of enzyme-immobilization methods, usually divided into numerous critical steps, and discrete optimization procedures of rational designs would possibly result in the introduction of strong and functional immobilized biocatalysts. A diagrammatic representation of the different methods of enzyme immobilization is summarized in Figure 1.

## Classification of Immobilized-Enzymes

Immobilized enzymes have been classified into two major groups that are given as follows:

A) Carrier-bound immobilized enzymes: These are the enzymes that are physically or chemically bound to a matrix or support (i.e., carrier).
B) Carrier-free immobilized enzymes do not need supererogatory inactive mass. Carrier-free immobilized enzymes are normally constructed on the basis of their molecular mass *via* chemical cross-linking (Cao, 2011).

## Need for Carrier-Free Immobilized Enzymes

In the past few decades, the use of an immobilized enzyme has become a major priority in industrial processes. The use of non-toxic, biodegradable, renewal, and commercially sustainable carrier-free immobilized enzymes and their physical or chemical property to fit in with its counterpart enzyme (or biocatalyst) makes it insoluble, aids during the separation process, and their continuous reusability in industrial or commercial processes (Sheldon, 2019; Ottone et al., 2020). Thompson et al. (2019a) have defined the parameters that an immobilized enzyme must satisfy in order to be commercially viable: easier recovery, more recyclability (∼above 20 cycles), stable during the reaction process, lower cost, tolerant to harsh solvents, minimum or no leaching, maximum activity recovery (∼above 50%), and maximum loading of the enzyme. Carrier-free immobilization as a cross-linked enzyme(s) and their derivatives is one way to do this. With a wide range of enzymes, particularly carbohydrate-converting enzymes, this technology is proved to be quite successful (Contesini et al., 2013).

The selection of good carriers gives clean control over the non-catalytic units of the acquired immobilized biocatalyst. The physical and the chemical nature of the carrier such as chemical composition, hydrophobic/hydrophilic balance, pore size, and binding chemistries dictates the performance of a carrier-bound immobilized enzyme (i.e., enzyme activity and stability) and a good carrier or suitable binding chemistry for an enzyme is not necessarily the right one for other enzymes or other applications (Sheldon, 2019). Thus, the nature of the selected carrier may be taken into consideration so as to modify the biocatalyst. Correspondingly, a high-quality quantity of artificial or organic/herbal carrier matrix, with unique shapes/sizes, porous/non-porous structures, and binding functionalities are particularly designed for diverse bio-immobilization and bio-separation procedures (Cao, 2011). Regardless of accelerated expertise on carrier-based enzyme immobilization, the layout of the carrier and certain immobilized enzymes nonetheless are based largely on rigorous screening procedures.

## Major Types of Carrier-Free Immobilized Enzymes

Carrier-free immobilized enzymes do not require additional inactive material or mass, that is, a carrier. At present, the following approaches have been devised for creating a carrier-free immobilized enzyme (Table 1), namely, cross-linked dissolved enzyme: CLEs; cross-linked enzyme crystals (CLECs); cross-linked enzyme aggregates (CLEAs); cross-linked enzyme lyophilizates (CLELs); and cross-linked spray-dried enzymes (CSDEs) (Wilson et al., 2004). Thus, utilizing various cross-linking precursors aids in distinguishing between different types of carrier-free immobilized enzymes.

**TABLE 1:** Comparison of different properties of soluble, carrier-bound immobilized, and carrier-free immobilized enzymes (Jegan Roy and Emilia Abraham, 2004; Cui et al., 2014; Voběrková et al., 2018).

Parameter	Soluble enzyme	Carrier-bound immobilized enzymes	Carrier-free immobilized enzymes			
			CLEs^a^	CLECs^a^	CLEAs^a^	CSDEs^a^
Purity level required for synthesis	Crude or purified enzyme	Crude or purified enzyme	Crude or purified enzyme	Only purified enzyme	Crude or purified enzyme	Purified enzyme only
Storage conditions	Refrigeration required	Refrigeration required	Can be refrigerated or stored at room temperature	Can be refrigerated or stored at room temperature	Can be refrigerated or stored at room temperature	Can be refrigerated or stored at room temperature
Activity	High activity	Reduced activity due to higher concentration of carrier	High activity due to increased volumetric activity	High activity due to increased volumetric activity	High activity due to increased volumetric activity	Limited activity due to drying of enzyme
Media	Aqueous	More reactive in aqueous and less in organic media	More reactive in both aqueous and organic media	More reactive in both aqueous and organic media	More reactive in both aqueous and organic media	More reactive in both aqueous and organic media
pH and thermo-stability	Limited pH and temperature range	Limited pH and temperature range	Stable pH and temperature range	Stable pH and temperature range	Stable pH and temperature range	Stable pH and temperature range
Processivity	Low	High	High	Very high	Very high	Low

a CLEs, cross-linked enzymes; CLECs, cross-linked enzyme crystals; CLEAs, cross-linked enzyme aggregates; CSDEs, cross-linked spray-dried enzymes.

The use of carriers in carrier-bound enzymes could decrease catalytic activity due to dilution of the enzyme due to the inclusion of more than 95 percent non-catalytic unit in the form of carrier (Roessl et al., 2010). For some applications, this might result in unacceptably reduced volumetric and space-time yields, as well as decreased catalyst efficiency. In contrast, carrier-free immobilized enzymes, particularly cross-linked enzyme aggregates and cross-linked enzyme crystals, perform well (DeSantis and Jones, 1999). As a result, significant research has been conducted to train these carrier-free immobilized enzymes, particularly CLEs. More than 20 different enzymes have been directly cross-connected to form many CLEs that were originally adsorbed on inert supports, including membranes cross-connected to shape supported CLEs (Cao, 2011).

### Cross-linking Enzymes

A dissolved enzyme may be cross-linked to increase its thermostability; however, additional factors that may influence the stability of such biocatalysts include the amount of cross-linker, temperature, ionic strength, pH, and the amount of dissolved enzyme used (Taylor, 1985).

Despite many improvements, it is very difficult to optimize stronger mechanical balance *via* CLEs entrapment or dissolved enzyme cross-linking in a gel matrix (Manecke, 1972). The usage of greater mass glaringly decreased the volumetric interest to the extent of a service-sure immobilized enzyme. Consequently, in many biocatalytic studies, scientists switched to carrier-bound enzymes with an extensive variety of carriers. Thus, many companies particularly exploited advanced immobilization techniques (Mosbach, 1971), and numerous reactions for binding enzymes to carriers were established (Cui and Jia, 2015).

### Cross-linking Enzymes Crystals

The remarkable discovery that is cross-linking of enzyme crystals of dissolved enzymes with a bifunctional chemical cross-linker, such as glutaraldehyde, could result in the formation of what we now refer to as insoluble CLECs, which was made in the early 1960s by researchers studying solid-phase protein chemistry by synthesizing compact cross-linked crystals of carboxypeptidase A (Quiocho and Richards, 1964). Following this work, a few other enzyme crystals were made using enzymes such as ribonuclease A, lysozyme (Manecke, 1972), subtilisin (Tüchsen and Ottesen, 1977, carboxypeptidase A (Quiocho and Richards, 1966), and alcohol dehydrogenase (Lee et al., 1986).

When compared to non-immobilized equivalents and standard carrier-bound immobilized enzymes (López–Serrano et al., 2002), their excellent stability under severe temperature and wider pH range in solvents made them an appealing prospective biocatalytic tools. Furthermore, it was demonstrated that CLECs could be designed in a reasonably short time because of no requirement of highly purified enzyme (Roessl et al., 2010). It was feasible to preserve comparable activity and selectivity relative to the soluble enzyme in an aqueous medium or relative to the crude enzyme in organic solvents by selecting the correct crystal shape or size or manipulating the crystallization characteristics of medium. The activity would also be affected by the size and characteristics of the substrates, the reaction media, the kind of reaction, and the reaction circumstances (Mehta et al., 2016). The biocatalytic activity could additionally depend upon the scale and residences of the substrates, the response medium, and response conditions (Cao et al., 1999). CLECs were formulated as strong and active immobilized enzymes of a controllable size. Several CLECs, particularly hydrolases consisting of acylases, proteases, and lipase, have been continuously used for chiral biocatalysis. Other uses of CLECs are microporous substances for controlled release of protein/peptide drugs, CLEC-based biosensor, lipase therapy for cystic fibrosis or pancreatitis, etc. (Jegan Roy and Emilia Abraham, 2004).

### Cross-Linking Enzyme Aggregates

**FIGURE 2:**
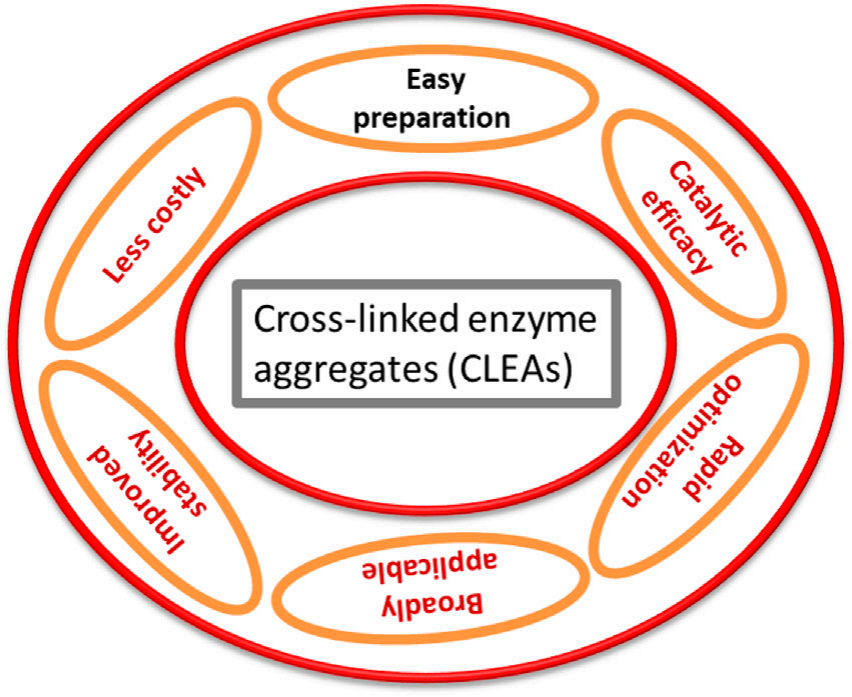
Features of cross-linked enzyme aggregates.

CLEAs have been introduced as one of most effective carrier-free immobilized enzyme systems (Figure 2), and the major advantage of this technique is that a tedious purification step is not required (Thompson et al., 2019b). By altering their properties that affect the proximity of soluble enzyme molecules, they can be used to shape bodily aggregates that, after cross-linking, termed as CLEAs (Cao et al., 2003; Table 2). When distributed in an aqueous media, these solid aggregates are kept together by non-covalent bonding and are easily collapsed and redissolved as a result of the non-covalent bonding. In the case of physical aggregates, chemical cross-linking would result in the formation of cross-linked enzyme aggregates, in which the restructured superstructure of the aggregates and their activity would be preserved (Nadar et al., 2016; Alves et al., 2021). This can partly explain why the enzyme could not consistently be cross-linked, even when 80% of overall lysine residues were changed through glutaraldehyde (Tomimatsu et al., 1971). Interestingly, it determined that the catalytic behavior of CLEAs differs because of the presence of the precipitants. In the case of CLEAs of penicillin G acylase produced through ammonium sulfate precipitation, the biocatalyst displayed similar behavior to the local enzyme for ampicillin synthesis, while CLEAs employed the use of *test*-butanol as a precipitant (Ling et al., 2016).

**TABLE 2:** Various types of support systems used in CLEAs.

Types of support system	Different classes of enzymes	Techniques utilized for immobilization	References
Super nanoporous silica	Lipase; chymotrypsin	Cross-linking and adsorption	Iyer and Ananthanarayan (2008)
Nanoporous silicate foam	Beta-glucosidase	Cross-linking and adsorption	Califano and Costantini (2020)
Silica gel (macroporous in nature)	Pepsin (papain)	Cross-linking and adsorption	Hudson et al. (2008)
Sol-gel system	Lipase (lipid-digesting)	Encapsulating	Acevedo-Fani et al. (2021)
Lentikats	Penicillin amidase	Encapsulating	Sawant et al. (2020)
Foaming agent monocellular in nature	Glucose–oxidase	Entrapping and cross-linking	Wahab et al. (2020)
Super nanoporous silica (SAB 15)	Lipase; chymotrypsin	Cross-linking and adsorption	Zhou and Hartmann (2013)
Hydro-gel pellet	Subtilisin	Entrapping	Jana et al. (2017)
Polylysine	Citrate synthase and subtilisin	Cross-linking	Yamaguchi et al. (2011)
Nano-fibers	Carbonic anhydrase	Cross-linking and adsorption	Ranimol et al. (2021)
Microporous polymeric sheet	Lipase (lipid digesting)	Embedded	Liu et al. (2021)
Chitosan electrospun	Lysozymes	Cross-linking and adsorption	Ribeiro et al. (2021)
Polystyrene nano-fibers	Lysozymes	Cross-linking and adsorption	Sabzehmeidani and Ghaedi (2021)
Magnetic nanoparticle	Esterases	Cross-linking	Sharma et al. (2021)
Mesocellular super nanoporous silicate	Chymotrypsin	Cross-linking	Vinu et al. (2008)
Calcium alginate (gelling form)	Tyrosinase	Encapsulating	Wei et al. (2021)

In most biological processes, more than one enzyme(s) are included, allowing them to maintain a high level of efficiency during metabolic and anabolic processes. In order to attain this aim during *in vitro* conditions, multiple-enzyme catalysis is preferred. Hence, combined cross-linked enzyme aggregates (combi-CLEAs) based on the CLEAs were designed and tested in the laboratory. Combi-CLEAs are enzyme complexes that include two or more immobilized enzymes that are capable of catalyzing sequential or simultaneous reactions in the same system (Sheldon, 2019).

Magnetic CLEA(s) of several enzymes has been synthesized by chemically cross-linking enzyme aggregates with magnetic nanoparticles, which can be isolated readily from the process mixture using a magnetic field (Sheldon, 2019). Moreover, wide-range thermostability increased buffering capacity, and varied pH has also been observed (Talekar et al., 2012).

### Cross-Linked Enzyme Lyophilizates

The cross-linked enzyme lyophilizates are synthesized from freeze-dried/lyophilized enzyme preparation in presence of lyoprotectants (polymers/sugars) to minimize denaturation during the drying stages, and these lyophilizates are subjected to cross-linkers and precipitants. Organic compounds, carbohydrates, amino acids, etc. and their derivatives can be produced using CLELs through enzymatic processes such as reduction, esterification, and asymmetric conversion processes (Jegan Roy and Emilia Abraham, 2004).

### Cross-Linked Spray-Dried Enzymes

Spray-dried enzyme powders are cross-linked to produce CSDEs. In addition, the fact that CSDEs are reversibly deactivated by the spray-drying process has prevented this technique from being widely used, even though it yielded respectable activity. Therefore, when compared to other carrier-free immobilization techniques, CSDEs have lower biocatalytic activity (Zicari et al., 2017).

## Process Optimization of Carrier-Free Enzymes

Immobilized enzyme preparations are effective biocatalysts for commercial manufacturing processes (Table 3). The introduction of carrier-free immobilized enzymes for process optimization has tackled major drawbacks of carrier bound immobilized enzymes by reducing the use of expensive carriers and increased catalytic mass with increased yield and reduced costs in the scale-up process (Cao et al., 2003). It is possible to achieve increased thermostability by cross-linking the dissolved enzyme (CLEs), but this needed a precise balance between numerous elements, including the quantity of cross-linkers used, temperature, pH, and ionic strength of the solution. Furthermore, intermolecular cross-linking of these highly solvated enzyme molecules often resulted in a number of undesirable side effects, including decreased activity retention, poor repeatability, and limited mechanical stability.

**TABLE 3:** Commercial uses of carrier free enzymes.

Enzyme	Source	Class	Type	Commercial use	References
Thermolysin	*Bacillus thermoproteolyticus*, *Bacillus* spp.	Protease	CLECs	Manufacturing of artificial sweetener aspartame	Clair and Navia (1992)
Trypsin	Pancreatic trypsin in vertebrates	Protease	CLEAs	Food processing industry, clinical use, biotechnological processes	Menfaatli and Zihnioglu (2015)
Rabbit muscle fructose diphosphate aldolase	Rabbit muscle	Aldolase	CLECs	Synthesis of euk. RNA pol inhibitor	Sobolov et al. (1994)
Papain	*Carica papaya* fruit	Protease	CLEs	Leather, cosmetic, textiles, detergents, food, and pharmaceutical industries	Jansen and Olson (1969)
Penicillin acylase	Bacteria, yeast, and fungi	Hydrolase	CLEAs	Production of beta-lactam antibiotics	Cao et al. (2000)
Lipase, esterase	*Pseudomonas stutzeri*, *Candida antarctica*, *Thermomyces lanuginosus*, *Rhizomucor miehei*, *Aspergillus niger*, *Mucor miehei*	Hydrolase	CLEAs, Magnetic-CLEAs, CLECs	Processing of fats and oils, detergents and degreasing formulations, food processing, the synthesis of fine chemicals and pharmaceuticals, paper manufacture, and production of cosmetics	Adam et al. (1999), Schoevaart et al. (2004), Paitaid and H-Kittikun (2020), Guajardo et al. (2021)
Nitrile hydratase; alkaliphilic nitrile hydratase	*N. alkaliphilus*	Lyase	CLEAs; combi-CLEAs	Acrylamide production; aldehydes to (S)-α-hydroxycarboxylic acid amides	van Pelt et al. (2009), Gao et al. (2015)
Hydroxynitrile lyase	*M. esculenta*	Lyase	Combi-CLEAs	Synthesis of agrochemicals	Roberge et al. (2007), Lanfranchi et al. (2015)
Subtilisin	*Bacillus subtilis*	Protease	CLEAs	Stain cutter, cosmetics, food processing, skincare ointments, contact lens cleaners	Sangeetha and Abraham (2008)
Acylase	Porcine kidney	Hydrolase	CLEAs	Used as antifouling agent which causes biofilm degradation (replacement of tributyltin)	Lee et al. (2017)
Alginate lyase	*Flavobacterium* sp.	Lyase	CLEAs	Degrading gel	Kunjukunju et al. (2018)
Xylanase	*B. licheniformis*	Hydrolase	CLEAs	Paper and pulp industry, food processing	Kumar et al. (2017)
Phenylalanine ammonia lyase	*Rhodotorula glutinis*	Lyase	CLEAs	Conversion of L-phenylalanine to ammonia and trans-cinnamic acid	Cui et al. (2014)
Laccase	*Coriolus versicolor*, *Trametes versicolor*, *Trametes villosa*, *Agaricus bisporus*	Oxidoreductase	CLEAs	Elimination of undesirable phenolic compounds in baking, juice processing, wine stabilization, and bioremediation of wastewater	Bourbonnais and Paice (1990), Matijošytė et al. (2010)
Glucose/xylose isomerase	*Streptomyces thermonitrificans*	Isomerase	Magnetic-CLEAs	High-fructose corn syrup	Gupta and Srivastava (2017)
Peroxidase	*Bjerkandera adusta*	Oxidoreductase	Combi-CLEAs	Pharmaceutical preparations, treatment of industrial wastes	Taboada-Puig et al. (2011)
Ligninolytic enzymes	*Trametes versicolor*	Oxidoreductases	Combi-CLEAs	Decolorizing ability	Li Y. et al. (2015)
Penicillin amidase	Recombinant *Escherichia coli*	Hydrolase	Combi-CLEAs	Ampicillin, 6-aminopenicillanic acid	Illanes et al. (2006)
Lactase	*Kluyveromyces lactis*	Hydrolase	CLEAs	Lactose-free milk	Dong and Zhong (2019), Wilson et al. (2022)
Cellulase–xylanase mixture	Microorganisms, algae, protozoans, crustaceans, and insects	Hydrolase	CSDEs	Fabric softening, pulp processing, bio-bleaching, oil extraction, beverage production, bioscouring	Santa-Maria et al. (2012)
Tyrosinase	Mushroom tyrosinase	Oxidoreductase	CLEAs	Elimination of phenolic compounds from wastewater	Xu and Yang (2013)
Carbonic anhydrase	*Rhodobacter sphaeroides*	Lyase	CLEAs	Carbon sequestration and biofuel production	Park et al. (2012)
Phytase	Soya milk	Hydrolase	CLEAs	Animal feed supplement	Tirunagari et al. (2018)
β-galactosidase	Recombinant *E. coli* BL21	Hydrolase	CLEAs	Synthesis of galacto-oligosaccharides	Li L. et al. (2015)
Monoamine oxidase	*Arthrobacter aurescens*	Oxidoreductase	CLEAs	Determination of biogenic monoamines	Kim and Kim (2016)
Transglutaminase	Plants, microbial origin	Transferase	CSDEs	Flavoring agent	Gong et al. (2019)
Urease	Jack bean	Hydrolase	CLELs	Fertilizers industry, clinical kits, reducing agents in beverages.	Akkas et al. (2020)
L-methioninase	Bacterial, fungal, and plant origin	Lyase	CLEAs	Therapeutic formulations	Kannan and Marudhamuthu (2019)
Amylase	*Bacillus lehensis* G1	Hydrolase	CLEAs, magnetic-CLEAs	Fuel alcohol production, detergent, textile, paper industry, starch conversion	Nadar et al. (2016), Nawawi et al. (2020)
Glycerol dehydrogenase and NADH oxidase	Recombinant *E. coli* BL21	Oxidoreductase	Combi-CLEAs	Synthesis of chiral chemicals	Xu et al. (2020)

Cross-linked enzyme crystals (CLECs) were introduced by Quiocho and Richards (1964). When compared to CLEs, it was discovered that CLECs demonstrated improved thermostability, pH, more tolerance to organic solvents, and mechanical forces and showed higher retained activity. But one major drawback to CLECs was the requirement of high purified enzymes and their crystallization, which makes this process costlier. This limitation was overcome by a more promising, commercially utilized technique, that is, CLEAs (Sheldon, 2011). It is synthesized in two different phases: The initial step includes enzyme aggregation by precipitants using methods such as salting out with ammonium sulfate, organic solvents, isoelectric precipitation by TCA, using polyethylenimine, etc. which is then followed by the establishment of chemical linkages between the enzymes via cross-linking agents such as glutaraldehyde to further strengthen the interactions. The aggregation of proteins is exploited by rapid change of their hydration state by the addition of precipitants in the solvent solution. The development of precipitated enzyme aggregates is a necessary step in the preservation of enzyme activity during cross-linking (Arana-Peña et al., 2021). It has been observed that the catalytic activity of CLEAs varies based on the characteristics of the precipitants used in aggregation. In addition, spray drying is a reasonably affordable and readily scaled-up approach that is repeatable, making it a useful method of encapsulation technologies in industrial processes (Cui and Jia, 2015). At present, CSDEs have limited use in the industrial process but are still a robust and emerging technique. The objective is to provide a highly adaptable technological platform for screening and building strong carrier-free enzymes for a wide range of commercial applications.

## Future Prospectus

In summary, the carrier-free immobilized enzyme technology has gained interest among researchers and engineers due to its commercial applicability in industrial processes. Many variants of cross-linked enzymes have proven their multiple applications, such as biotransformation processes, water treatment, antibiotic production, food processing, and several other potential applications. Another advantage in commercial use is their primary preference to replace toxic compounds in future chemical industries with more ecofriendly biocatalytic enzymes. Combi-CLEAs and magnetic CLEAs have proven to be more convenient in future production processes due to the presence of multiple catalysts in individual aggregates and easier separation.

## Conclusion

Although the selected approach of immobilization may differ from enzyme to enzyme, carrier to carrier, and for a different application, primarily relying upon the peculiarities of every unique process, standards for measuring immobilized enzyme’s robustness remain the same. Commercially applied immobilized enzymes need to be relatively active, relatively selective (to lessen cross-reactions), relatively stable (to lessen value *via* way of means of efficient reuse), value-intensive (low-value contribution therefore economically viable), secure to use (to satisfy protection regulations), and definitely innovative. The productiveness of almost every immobilized enzyme is relatively lower than that of chemical processes. Due to diffusion constraints, activity retention for porous carriers is regularly underneath 50% at most enzyme loading in a biocatalytic reaction system. Although improvement of carrier-free enzymes, including CLEA or CLEC, can put off the use of the non-catalytic mass provider, the intrinsic drawbacks related to the carrier-free immobilized enzymes. The carrier-free biocatalytic systems appear to be greatly appealing as no scaffold/matrix is required, no matrix modification or activation is needed, little leaching effect is seen, and the complete absence of aldehyde cross-linking chemicals of the benefits of such biocatalytic systems. With the progressive research in the field, the future seems to be bright in creating advanced techniques to immobilize different enzymes, which would result in enhancing the efficiency of the enzyme by many folds.

## References

[B1] Acevedo-Fani A. Guo Q. Nasef N. Singh H. (2021). “Aspects of Food Structure in Digestion and Bioavailability of LCn-3PUFA-Rich Lipids,” in Omega-3 Delivery Systems (Academic Press), 427–448. 10.1016/b978-0-12-821391-9.00003-x

[B2] Adam W. Lazarus M. Saha-Möller C. R. Weichold O. Hoch U. Häring D. (1999). Biotransformations with Peroxidases. Adv. Biochem. Eng. Biotechnol. 63, 73–108. 10.1007/3-540-69791-8_4 9933982

[B3] Ahmad R. Sardar M. (2015). Enzyme Immobilization: An Overview on Nanoparticles as Immobilization Matrix. Biochem. Anal. Biochem. 4 (2), 1. 10.4172/2161-1009.1000178

[B4] Akkas T. Zakharyuta A. Taralp A. Ow-Yang C. W. (2020). Cross-Linked Enzyme Lyophilisates (CLELs) of Urease: A New Method to Immobilize Ureases. Enzyme Microb. Technol. 132, 109390. 10.1016/j.enzmictec.2019.109390 31731959

[B5] Almeida P. Z. Messias J. M. Pereira M. G. Pinheiro V. E. Monteiro L. M. O. Heinen P. R. (2018). Mixture Design of Starchy Substrates Hydrolysis by an Immobilized Glucoamylase fromAspergillus Brasiliensis. Biocatal. Biotransform. 36 (5), 389395–395. 10.1080/10242422.2017.1423059

[B6] Alves N. R. Pereira M. M. Giordano R. L. C. Tardioli P. W. Lima Á. S. Soares C. M. F. (2021). Design for Preparation of More Active Cross-Linked Enzyme Aggregates of Burkholderia Cepacia Lipase Using palm Fiber Residue. Bioproc. Biosyst Eng. 44 (1), 57–66. 10.1007/s00449-020-02419-0 32767112

[B7] Arana-Peña S. Carballares D. Morellon-Sterlling R. Berenguer-Murcia Á. Alcántara A. R. Rodrigues R. C. Fernandez-Lafuente R. (2021). Enzyme Co-Immobilization: Always the Biocatalyst Designers’ Choice…or Not? 51. 10.1016/j.biotechadv.2020.107584 32668324

[B8] Bourbonnais R. Paice M. G. (1990). Oxidation of Non-phenolic Substrates: An Expanded Role for Laccase in Lignin Biodegradation. Biotechnol. Adv. 267 (1), 107584–108102. 10.1016/0014-5793(90)80298-w 2365094

[B9] Califano V. Costantini A. (2020). Immobilization of Cellulolytic Enzymes in Mesostructured Silica Materials. Catalysts 10 (6), 706. 10.3390/catal10060706

[B10] Cao L. (2011). “Immobilized Enzymes,” in Immobilized Enzymes. Comprehensive Biotechnology. Editor Moo-Young M.. 2nd Edn. (Academic Press), 461–476. 10.1016/B978-0-08-088504-9.00168-9

[B11] Cao L. (2005). “Introduction: Immobilized Enzymes: Past, Present, and Prospects,” in Carrier-Bound immobilized enzymes: Principles, Application, and Design (Weinheim: Wiley‐VCH Verlag GmbH & Co. KGaA), 1–52.

[B12] Cao L. Langen L. v. Sheldon R. A. (2003). Immobilised Enzymes: Carrier-Bound or Carrier-free? Curr. Opin. Biotechnol. 14 (4), 387–394. 10.1016/s0958-1669(03)00096-x 12943847

[B13] Cao L. Van Langen L. Janssen M. Sheldon R. (1999). Preparation and Properties of Cross-Linked Aggregates of Penicillin Acylase and Other Enzymes. European Patent EP1088887A1. Munich, Germany: European Patent Office.

[B14] Cao L. van Rantwijk F. Sheldon R. A. (2000). Cross-linked Enzyme Aggregates: A Simple and Effective Method for the Immobilization of Penicillin Acylase. Org. Lett. 2 (10), 1361–1364. 10.1021/ol005593x 10814447

[B15] Cao Y. Li X. Ge J. (2021). Enzyme Catalyst Engineering toward the Integration of Biocatalysis and Chemocatalysis. Trends Biotechnol. 39 (11), 1173–1183. 10.1016/j.tibtech.2021.01.002 33551176

[B16] Chapman J. Ismail A. Dinu C. (2018). Industrial Applications of Enzymes: Recent Advances, Techniques, and Outlooks. Catalysts 8 (6), 238. 10.3390/catal8060238

[B17] Contesini F. de Alencar Figueira J. Kawaguti H. de Barros Fernandes P. de Oliveira Carvalho P. da Graça Nascimento M. (2013). Potential Applications of Carbohydrases Immobilization in the Food Industry. Int. J. Mol. Sci. 14 (1), 1335–1369. 10.3390/ijms14011335 23344046 PMC3565324

[B18] Cui J. d. Cui L. l. Zhang S. p. Zhang Y. f. Su Z. g. Ma G. h. (2014). Hybrid Magnetic Cross-Linked Enzyme Aggregates of Phenylalanine Ammonia Lyase from Rhodotorula Glutinis. PLoS One 9 (5), e97221. 10.1371/journal.pone.0097221 24825453 PMC4019550

[B19] Cui J. D. Jia S. R. (2015). Optimization Protocols and Improved Strategies of Cross-Linked Enzyme Aggregates Technology: Current Development and Future Challenges. Crit. Rev. Biotechnol. 35 (1), 15–28. 10.3109/07388551.2013.795516 23886350

[B20] Datta S. Christena L. R. Rajaram Y. R. S. (2013). Enzyme Immobilization: An Overview on Techniques and Support Materials. 3 Biotech. 3 (1), 1–9. 10.1007/s13205-012-0071-7 PMC3563746 28324347

[B21] DeSantis G. Jones J. B. (1999). Chemical Modification of Enzymes for Enhanced Functionality. Curr. Opin. Biotechnol. 10 (4), 324–330. 10.1016/S0958-1669(99)80059-7 10449313

[B22] Dong L. Zhong Q. (2019). Dispersible Biopolymer Particles Loaded with Lactase as a Potential Delivery System to Control Lactose Hydrolysis in Milk. J. Agric. Food Chem. 67 (23), 6559–6568. 10.1021/acs.jafc.9b01546 31099562

[B23] Dwevedi A. (2016). Enzyme Immobilization: Advances in Industry, Agriculture, Medicine, and the Environment. Switzerland: Springer International Publishing.

[B24] Facchini F. Pereira M. Vici A. Filice M. Pessela B. Guisan J. (2018). Immobilization Effects on the Catalytic Properties of Two Fusarium Verticillioides Lipases: Stability, Hydrolysis, Transesterification and Enantioselectivity Improvement. Catalysts 8 (2), 84. 10.3390/catal8020084

[B25] Gao J. Wang Q. Jiang Y. Gao J. Liu Z. Zhou L. (2015). Formation of Nitrile Hydratase Cross-Linked Enzyme Aggregates in Mesoporous Onion-like Silica: Preparation and Catalytic Properties. Ind. Eng. Chem. Res. 54 (1), 83–90. 10.1021/ie503018m

[B26] Gong P. Di W. Yi H. Sun J. Zhang L. Han X. (2019). Improved Viability of spray-dried Lactobacillus Bulgaricus sp1.1 Embedded in Acidic-Basic Proteins Treated with Transglutaminase. Food Chem. 281, 204–212. 10.1016/j.foodchem.2018.12.095 30658749

[B27] Guajardo N. Ahumada K. Domínguez de María P. (2021). Immobilization of Pseudomonas Stutzeri Lipase through Cross-Linking Aggregates (CLEA) for Reactions in Deep Eutectic Solvents. J. Biotechnol. 337, 18–23. 10.1016/j.jbiotec.2021.06.021 34171440

[B28] Gupta A. Srivastava S. K. (2017). Study of Cross Linked Enzyme Aggregate of Glucose Isomerase of Streptomyces Thermonitrificans Immoblised on Magnetic Particle. J. Biochem. Technol. 7 (1), 1102–1106.

[B29] Heinen P. R. Pereira M. G. Rechia C. G. V. Almeida P. Z. Monteiro L. M. O. Pasin T. M. (2017). Immobilized Endo-Xylanase of Aspergillus tamarii Kita: An Interesting Biological Tool for Production of Xylooligosaccharides at High Temperatures. Process Biochem. 53, 145–152. 10.1016/j.procbio.2016.11.021

[B30] Hudson S. Cooney J. Magner E. (2008). Proteins in Mesoporous Silicates. Angew. Chem. Int. Ed. 47 (45), 8582–8594. 10.1002/anie.200705238 18833554

[B31] Illanes A. Wilson L. Caballero E. Fernández-Lafuente R. Guisán J. M. (2006). Crosslinked Penicillin Acylase Aggregates for Synthesis of β-Lactam Antibiotics in Organic Medium. Abab 133 (3), 189–202. 10.1385/ABAB:133:3:189 16720900

[B32] Iyer P. V. Ananthanarayan L. (2008). Enzyme Stability and Stabilization-Aqueous and Non-aqueous Environment. Process Biochem. 43 (10), 1019–1032. 10.1016/j.procbio.2008.06.004

[B33] Jana S. Sen K. Gandhi A. Jana S. Roy C. (2017). “Role of Alginate in Drug Delivery Applications,” in Industrial Applications of marine Biopolymers (Florida: CRC Press), 369–399. 10.1201/9781315313535-18

[B34] Jansen E. F. Olson A. C. (1969). Properties and Enzymatic Activities of Papain Insolubilized with Glutaraldehyde. Arch. Biochem. Biophys. 129 (1), 221–227. 10.1016/0003-9861(69)90169-6 5762964

[B35] Jegan Roy J. Emilia Abraham T. (2004). September 8)Strategies in Making Cross-Linked Enzyme Crystals. Chem. Rev. 104 (9), 3705–3722. 10.1021/cr0204707 15352777

[B36] Kannan S. Marudhamuthu M. (2019). Development of Chitin Cross-Linked Enzyme Aggregates of L-Methioninase for Upgraded Activity, Permanence and Application as Efficient Therapeutic Formulations. Int. J. Biol. Macromol. 141, 218–231. 10.1016/j.ijbiomac.2019.08.246 31479672

[B37] Kim Y.-J. Kim Y.-W. (2016). Optimizing the Preparation Conditions and Characterization of Cross-Linked Enzyme Aggregates of a Monoamine Oxidase. Food Sci. Biotechnol. 25 (5), 1421–1425. 10.1007/s10068-016-0221-5 30263425 PMC6049291

[B38] Kricka L. J. Thorpe G. H. G. (1986). Immobilized Enzymes in Analysis. Trends Biotechnol. 4 (10), 253–258. 10.1016/0167-7799(86)90188-5

[B39] Kumar S. Haq I. Prakash J. Raj A. (2017). Improved Enzyme Properties upon Glutaraldehyde Cross-Linking of Alginate Entrapped Xylanase from Bacillus Licheniformis. Int. J. Biol. Macromol. 98, 24–33. 10.1016/j.ijbiomac.2017.01.104 28130131

[B40] Kunjukunju S. Roy A. Shekhar S. Kumta P. N. (2018). Cross-linked Enzyme Aggregates of Alginate Lyase: A Systematic Engineered Approach to Controlled Degradation of Alginate Hydrogel. Int. J. Biol. Macromol. 115, 176–184. 10.1016/j.ijbiomac.2018.03.110 29578011

[B106] Lanfranchi E. Winkler M. Pavkov-Keller T. Köhler E. M. Diepold M. Darnhofer B. (2015). “Hydroxynitrile Lyase from Fern: A Unique Biocatalyst,” in 16th Austrian Chemistry Days 2015 (Innsbruck, Austria).

[B41] Lee J. Lee I. Nam J. Hwang D. S. Yeon K.-M. Kim J. (2017). Immobilization and Stabilization of Acylase on Carboxylated Polyaniline Nanofibers for Highly Effective Antifouling Application via Quorum Quenching. ACS Appl. Mater. Inter. 9 (18), 15424–15432. 10.1021/acsami.7b01528 28414213

[B42] Lee K. M. Blaghen M. Samama J.-P. Biellmann J.-F. (1986). Crosslinked Crystalline Horse Liver Alcohol Dehydrogenase as a Redox Catalyst: Activity and Stability toward Organic Solvent. Bioorg. Chem. 14 (2), 202–210. 10.1016/0045-2068(86)90031-3

[B43] Li L. Li G. Cao L.-c. Ren G.-h. Kong W. Wang S.-d. (2015). Characterization of the Cross-Linked Enzyme Aggregates of a Novel β-Galactosidase, a Potential Catalyst for the Synthesis of Galacto-Oligosaccharides. J. Agric. Food Chem. 63 (3), 894–901. 10.1021/jf504473k 25557319

[B44] Li Y. Wang Z. Xu X. Jin L. (2015). A Ca-Alginate Particle Co-immobilized with Phanerochaete Chrysosporium Cells and the Combined Cross-Linked Enzyme Aggregates from *Trametes versicolor* . Bioresour. Technol. 198, 464–469. 10.1016/j.biortech.2015.09.032 26413897

[B45] Ling X.-M. Wang X.-Y. Ma P. Yang Y. Qin J.-M. Zhang X.-J. (2016). Covalent Immobilization of Penicillin G Acylase onto Fe3O4@Chitosan Magnetic Nanoparticles. J. Microbiol. Biotechnol. 26 (5), 829–836. 10.4014/jmb.1511.11052 26869599

[B46] Liu J. Ma R.-T. Shi Y.-P. (2020). “Recent Advances on Support Materials for Lipase Immobilization and Applicability as Biocatalysts in Inhibitors Screening Methods”-A Review. Analytica Chim. Acta 1101, 9–22. 10.1016/j.aca.2019.11.073 32029123

[B47] Liu L.-H. Chiu R.-Y. So P. B. Lirio S. Huang H.-Y. Liu W.-L. (2021). Fragmented α-Amylase into Microporous Metal-Organic Frameworks as Bioreactors. Materials 14 (4), 870. 10.3390/ma14040870 33670380 PMC7918099

[B48] López-Serrano P. Cao L. Van Rantwijk F. Sheldon R. A. (2002). Cross-linked Enzyme Aggregates with Enhanced Activity: Application to Lipases. Biotechnol. Lett. 24 (16), 1379–1383. 10.1023/A:1019863314646

[B49] Manecke G. (1972). “Immobilization of Enzymes by Various Synthetic Polymers,” in Biotechnology and Bioengineering Symposium (New York: John Wiley & Sons), Vol. 3.

[B50] Matijošytė I. Arends I. W. C. E. de Vries S. Sheldon R. A. (2010). Preparation and Use of Cross-Linked Enzyme Aggregates (CLEAs) of Laccases. J. Mol. Catal. B Enzym. 62 (2), 142–148. 10.1016/j.molcatb.2009.09.019

[B51] Mehta J. Bhardwaj N. Bhardwaj S. K. Kim K.-H. Deep A. (2016). Recent Advances in Enzyme Immobilization Techniques: Metal-Organic Frameworks as Novel Substrates. Coord. Chem. Rev. 322, 30–40. 10.1016/j.ccr.2016.05.007

[B52] Menfaatli E. Zihnioglu F. (2015). Carrier Free Immobilization and Characterization of Trypsin. Artif. Cell Nanomed. Biotechnol. 43 (2), 140–144. 10.3109/21691401.2013.853178 24195581

[B53] Mohamad N. R. Marzuki N. H. C. Buang N. A. Huyop F. Wahab R. A. (2015). An Overview of Technologies for Immobilization of Enzymes and Surface Analysis Techniques for Immobilized Enzymes. Biotechnol. Biotechnol. Equip. 29 (2), 205–220. 10.1080/13102818.2015.1008192 26019635 PMC4434042

[B54] Monteiro L. M. O. Pereira M. G. Vici A. C. Heinen P. R. Buckeridge M. S. Polizeli M. d. L. T. d. M. (2019). Efficient Hydrolysis of Wine and Grape Juice Anthocyanins by Malbranchea Pulchella β-glucosidase Immobilized on MANAE-Agarose and ConA-Sepharose Supports. Int. J. Biol. Macromol. 136, 1133–1141. 10.1016/j.ijbiomac.2019.06.106 31220494

[B55] Mosbach K. (1976). Immobilised Enxymes. FEBS Lett. 62 (Suppl), E80–E94. 10.1016/0014-5793(76)80856-3 3436

[B56] Mosbach K. (1971). Enzymes Bound to Artificial Matrixes. Sci. Am. 224 (3), 26–33. 10.1038/scientificamerican0371-26 5546816

[B57] Nadar S. S. Muley A. B. Ladole M. R. Joshi P. U. (2016). Macromolecular Cross-Linked Enzyme Aggregates (M-CLEAs) of α-amylase. Int. J. Biol. Macromol. 84, 69–78. 10.1016/j.ijbiomac.2015.11.082 26675136

[B58] Nawawi N. N. Hashim Z. Manas N. H. A. Azelee N. I. W. Illias R. M. (2020). A Porous-Cross Linked Enzyme Aggregates of Maltogenic Amylase from Bacillus Lehensis G1: Robust Biocatalyst with Improved Stability and Substrate Diffusion. Int. J. Biol. Macromolecules 148, 1222–1231. 10.1016/j.ijbiomac.2019.10.101 31759025

[B59] Ottone C. Romero O. Aburto C. Illanes A. Wilson L. (2020). Biocatalysis in the Winemaking Industry: Challenges and Opportunities for Immobilized Enzymes. Compr. Rev. Food Sci. Food Saf. 19 (2), 595–621. 10.1111/1541-4337.12538 33325181

[B60] Paitaid P. H-Kittikun A. (2020). Magnetic Cross-Linked Enzyme Aggregates of Aspergillus oryzae ST11 Lipase Using Polyacrylonitrile Coated Magnetic Nanoparticles for Biodiesel Production. Appl. Biochem. Biotechnol. 190 (4), 1319–1332. 10.1007/s12010-019-03196-7 31754983

[B61] Palmer T. Bonner P. L. (2007). Enzymes: Biochemistry, Biotechnology, Clinical Chemistry. Amsterdam, Netherlands: Elsevier Science.

[B62] Park J.-M. Kim M. Lee H. J. Jang A. Min J. Kim Y.-H. (2012). Enhancing the Production of Rhodobacter Sphaeroides-Derived Physiologically Active Substances Using Carbonic Anhydrase-Immobilized Electrospun Nanofibers. Biomacromolecules 13 (11), 3780–3786. 10.1021/bm3012264 22988895

[B63] Patel A. B. Pennington S. N. Brown H. D. (1969). Insoluble Matrix-Supported Apyrase, Deoxyribonuclease and Cholinesterase. Biochim. Biophys. Acta Enzymol. 178 (3), 626–629. 10.1016/0005-2744(69)90232-0 4239443

[B64] Quiocho F. A. Richards F. M. (1964). Intermolecular Cross Linking of a Protein in the Crystalline State: Carboxypeptidase-A. Proc. Natl. Acad. Sci. 52, 833–839. 10.1073/pnas.52.3.833 14212562 PMC300354

[B65] Quiocho F. A. Richards F. M. (1966). The Enzymic Behavior of Carboxypeptidase-A in the Solid State*. Biochemistry 5 (12), 4062–4076. 10.1021/bi00876a041

[B66] Ranimol G. Paul C. Sunkar S. (2021). Optimization and Efficacy Studies of Laccase Immobilized on Zein-Polyvinyl Pyrrolidone Nano Fibrous Membrane in Decolorization of Acid Red 1. Water Sci. Technol. 84 (10–11), 2703–2717. 10.2166/wst.2021.200 34850688

[B67] Ray R. R. Jana S. C. Nanda G. (1994). Biochemical Approaches of Increasing Thermostability of β-amylase fromBacillus megateriumB6. FEBS Lett. 356 (1), 30–32. 10.1016/0014-5793(94)01227-x 7988714

[B68] Ribeiro E. S. de Farias B. S. Sant’Anna Cadaval Junior T. R. de Almeida Pinto L. A. Diaz P. S. (2021). Chitosan-based Nanofibers for Enzyme Immobilization. Int. J. Biol. Macromol. 183, 1959–1970. 10.1016/j.ijbiomac.2021.05.214 34090851

[B69] Roberge C. Fleitz F. Pollard D. Devine P. (2007). Asymmetric Synthesis of Cyanohydrin Derived from Pyridine Aldehyde with Cross-Linked Aggregates of Hydroxynitrile Lyases. Tetrahedron Lett. 48 (8), 1473–1477. 10.1016/j.tetlet.2006.12.053

[B70] Roessl U. Nahálka J. Nidetzky B. (2010). Carrier-free Immobilized Enzymes for Biocatalysis. Biotechnol. Lett. 32 (3), 341–350. 10.1007/s10529-009-0173-4 19943180

[B72] Sabzehmeidani M. M. Ghaedi M. (2021). “Adsorbents Based on Nanofibers,” in Interface Science and Technology (Amsterdam, Netherlands: Elsevier Science), 389–443. 10.1016/B978-0-12-818805-7.00005-9

[B73] Sangeetha K. Emilia Abraham T. (2008). Preparation and Characterization of Cross-Linked Enzyme Aggregates (CLEA) of Subtilisin for Controlled Release Applications. Int. J. Biol. Macromol. 43 (3), 314–319. 10.1016/j.ijbiomac.2008.07.001 18662715

[B74] Santa-Maria M. Scher H. Jeoh T. (2012). Microencapsulation of Bioactives in Cross-Linked Alginate Matrices by spray Drying. J. Microencapsul. 29 (3), 286–295. 10.3109/02652048.2011.651494 22251237

[B75] Sawant A. M. Sunder A. V. Vamkudoth K. R. Ramasamy S. Pundle A. (2020). Process Development for 6-aminopenicillanic Acid Production Using Lentikats-Encapsulated *Escherichia coli* Cells Expressing Penicillin V Acylase. ACS Omega 5 (45), 28972–28976. 10.1021/acsomega.0c02813 33225127 PMC7675567

[B76] Schoevaart R. Wolbers M. W. Golubovic M. Ottens M. Kieboom A. P. G. Van Rantwijk F. (2004). Preparation, Optimization, and Structures of Cross-Linked Enzyme Aggregates (CLEAs). Biotechnol. Bioeng. 87 (6), 754–762. 10.1002/bit.20184 15329933

[B77] Sharma D. Bhardwaj K. K. Gupta R. (2021). Immobilization and Applications of Esterases. Biocatal. Biotransform., 1–16. 10.1080/10242422.2021.2013825

[B78] Sheldon R. A. (2011). Cross-linked Enzyme Aggregates as Industrial Biocatalysts. Org. Process. Res. Dev. 15 (1), 213–223. 10.1021/op100289f

[B79] Sheldon R. A. Woodley J. M. (2018). Role of Biocatalysis in Sustainable Chemistry. Chem. Rev. 118 (2), 801–838. 10.1021/acs.chemrev.7b00203 28876904

[B80] Sheldon R. (2019). CLEAs, Combi-CLEAs and 'Smart' Magnetic CLEAs: Biocatalysis in a Bio-Based Economy. Catalysts 9 (3), 261. 10.3390/catal9030261

[B81] Sobolov S. B. Bartoszko-Malik A. Oeschger T. R. Montelbano M. M. (1994). Cross-linked Enzyme Crystals of Fructose Diphosphate Aldolase: Development as a Biocatalyst for Synthesis. Tetrahedron Lett. 35 (42), 7751–7754. 10.1016/0040-4039(94)80109-6

[B82] St. Clair N. L. Navia M. A. (1992). Cross-linked Enzyme Crystals as Robust Biocatalysts. J. Am. Chem. Soc. 114 (18), 7314–7316. 10.1021/ja00044a064

[B83] Taboada-Puig R. Junghanns C. Demarche P. Moreira M. T. Feijoo G. Lema J. M. (2011). Combined Cross-Linked Enzyme Aggregates from Versatile Peroxidase and Glucose Oxidase: Production, Partial Characterization and Application for the Elimination of Endocrine Disruptors. Bioresour. Technol. 102 (11), 6593–6599. 10.1016/j.biortech.2011.03.018 21504845

[B84] Talekar S. Ghodake V. Ghotage T. Rathod P. Deshmukh P. Nadar S. (2012). Novel Magnetic Cross-Linked Enzyme Aggregates (Magnetic CLEAs) of Alpha Amylase. Bioresour. Technol. 123, 542–547. 10.1016/j.biortech.2012.07.044 22944488

[B85] Taylor R. F. (1985). A Comparison of Various Commercially-Available Liquid Chromatographic Supports for Immobilization of Enzymes and Immunoglobulins. Analytica Chim. Acta 172, 241–248. 10.1016/S0003-2670(00)82611-2

[B86] Thompson M. P. Derrington S. R. Heath R. S. Porter J. L. Mangas-Sanchez J. Devine P. N. (2019a). A Generic Platform for the Immobilisation of Engineered Biocatalysts. Tetrahedron 75 (3), 327–334. 10.1016/j.tet.2018.12.004

[B87] Thompson M. P. Peñafiel I. Cosgrove S. C. Turner N. J. (2019b). Biocatalysis Using Immobilized Enzymes in Continuous Flow for the Synthesis of fine Chemicals. Org. Process. Res. Dev. 23 (1), 9–18. 10.1021/acs.oprd.8b00305

[B88] Tirunagari H. Basetty S. Rode H. B. Fadnavis N. W. (2018). Crosslinked Enzyme Aggregates (CLEA) of Phytase with Soymilk Proteins. J. Biotechnol. 282, 67–69. 10.1016/j.jbiotec.2018.07.003 29981446

[B89] Tomimatsu Y. Jansen E. F. Gaffield W. Olson A. C. (1971). Physical Chemical Observations on the α-chymotrypsin Glutaraldehyde System during Formation of an Insoluble Derivative. J. Colloid Interf. Sci. 36 (1), 51–64. 10.1016/0021-9797(71)90239-6

[B90] Tüchsen E. Ottesen M. (1977). Kinetic Properties of Subtilisin Type Carlsberg in the Crystalline State. Carlsberg Res. Commun. 42 (5), 407–420. 10.1007/BF02906125

[B91] van Pelt S. van Rantwijk F. Sheldon R. A. (2009). Synthesis of Aliphatic (S)-α-Hydroxycarboxylic Amides Using a One-Pot Bienzymatic Cascade of Immobilised Oxynitrilase and Nitrile Hydratase. Adv. Synth. Catal. 351 (3), 397–404. 10.1002/adsc.200800625

[B92] Velasco-Lozano S. López-Gallego F. Mateos-Díaz J. C. Favela-Torres E. (2016). Cross-linked Enzyme Aggregates (CLEA) in Enzyme Improvement - A Review. Biocatalysis 1 (1), 166–177. 10.1515/boca-2015-0012

[B93] Vinu A. Gokulakrishnan N. Mori T. Ariga K. (2008). “Bio‐inorganic Hybrid Nanomaterials,” in Structured Materials. Bio-Inorganic Hybrid Nanomaterials: Strategies, Synthesis, Characterization and Applications. 10.1002/9783527621446

[B94] Voběrková S. Solčány V. Vršanská M. Adam V. (2018). Immobilization of Ligninolytic Enzymes from white-rot Fungi in Cross-Linked Aggregates. Chemosphere 202, 694–707. 10.1016/j.chemosphere.2018.03.088 29602102

[B95] Wahab R. A. Elias N. Abdullah F. Ghoshal S. K. (2020). On the Taught New Tricks of Enzymes Immobilization: An All-Inclusive Overview. Reactive Funct. Polym. 152, 104613. 10.1016/j.reactfunctpolym.2020.104613

[B96] Watanabe S. Shimizu Y. Teramatsu T. Murachi T. Hino T. (1988). [48] Application of Immobilized Enzymes for Biomaterials Used in Surgery. Methods Enzymol. 137, 545–551. 10.1016/0076-6879(88)37050-3 3374358

[B97] Wei C. M. Feng C. Y. Li S. Zou Y. Yang Z. (2021). Mushroom Tyrosinase Immobilized in Metal-Organic Frameworks as an Excellent Catalyst for Both Catecholic Product Synthesis and Phenolic Wastewater Treatment. J. Chem. Technol. Biotechnol. 10.1002/jctb.6984

[B98] Wilson L. Illanes A. Ottone C. Romero O. (2022). Co-immobilized Carrier-free Enzymes for Lactose Upgrading. Curr. Opin. Green Sustain. Chem. 33, 100553. 10.1016/j.cogsc.2021.100553

[B99] Wilson L. Illanes A. Pessela B. C. C. Abian O. Fernández-Lafuente R. Guisán J. M. (2004). Encapsulation of Crosslinked Penicillin G Acylase Aggregates in Lentikats: Evaluation of a Novel Biocatalyst in Organic media. Biotechnol. Bioeng. 86 (5), 558–562. 10.1002/bit.20107 15129439

[B100] Xu D.-Y. Yang Z. (2013). Cross-linked Tyrosinase Aggregates for Elimination of Phenolic Compounds from Wastewater. Chemosphere 92 (4), 391–398. 10.1016/j.chemosphere.2012.12.076 23411085

[B101] Xu M.-Q. Li F.-L. Yu W.-Q. Li R.-F. Zhang Y.-W. (2020). Combined Cross-Linked Enzyme Aggregates of Glycerol Dehydrogenase and NADH Oxidase for High Efficiency *In Situ* NAD+ Regeneration. Int. J. Biol. Macromol. 144, 1013–1021. 10.1016/j.ijbiomac.2019.09.178 31669469

[B102] Yamaguchi H. Miyazaki M. Asanomi Y. Maeda H. (2011). Poly-lysine Supported Cross-Linked Enzyme Aggregates with Efficient Enzymatic Activity and High Operational Stability. Catal. Sci. Technol. 1 (7), 1256–1261. 10.1039/c1cy00084e

[B103] Zhang B. Weng Y. Xu H. Mao Z. (2012). Enzyme Immobilization for Biodiesel Production. Appl. Microbiol. Biotechnol. 93 (1), 61–70. 10.1007/s00253-011-3672-x 22083277

[B104] Zhou Z. Hartmann M. (2013). Progress in Enzyme Immobilization in Ordered Mesoporous Materials and Related Applications. Chem. Soc. Rev. 42 (9), 3894–3912. 10.1039/c3cs60059a 23570038

[B105] Zicari T. J. Scher H. B. Santa-Maria M. C. Strobel S. (2017). Spray Dry Method for Encapsulation of Biological Moieties and Chemicals in Polymers Cross-Linked by Multivalent Ions for Controlled Release Applications. U.S. Patent 9 (700), 519.

